# Unicentric Castleman's Disease Masquerading Pancreatic Neoplasm

**DOI:** 10.1155/2012/793403

**Published:** 2012-11-25

**Authors:** Saurabh Jain, Souvik Chatterjee, Jyoti Ranjan Swain, Pritha Rakshit, Partha Chakraborty, Santanu Sinha

**Affiliations:** ^1^Department of General Surgery, Medical College Hospital, Kolkata, 88 College Street, Kolkata 700073, India; ^2^Department of Plastic & Reconstructive Surgery, IPGMER, 244 Acharya Jagadish Chandra Bose Road, Kolkata 700020, India; ^3^Department of Pediatric Surgery, Medical College Hospital, Kolkata, 88 College Street, Kolkata 700073, India

## Abstract

Castleman's disease is a rare nonclonal proliferative disorder of the lymph nodes with an unknown etiology. Common locations of Castleman's disease are mediastinum, neck, axilla, and abdomen. Castleman's disease of a peripancreatic location masquerading as pancreatic neoplasm is an even rarer entity. On search of published data, we came across about 17 cases published on peripancreatic Castleman's disease until now. Here we are reporting a case of retropancreatic Castleman's disease masquerading as retroperitoneal neoplasm in a 46-year-old male patient.

## 1. Introduction

Castleman's disease (CD) or angiofollicular lymph node hyperplasia was first described by Castleman and Towne in 1954 as an asymptomatic benign hyperplastic lymph node disorder resembling a thymoma [1, 2]. It is a rare benign lymphoproliferative disorder of unknown etiology.

The most common location of Castleman's disease is mediastinum (70%) but involvement of extrathoracic sites has also been reported, for example, neck, axilla, pelvis and retroperitoneum [3], anywhere along the lymphatic chain. Retroperitoneal Castleman's disease in the peripancreatic region is a rare entity. 

## 2. Case Report

 A 46-year-old male patient presented with chief complaints of left upper quadrant pain abdomen for last 3 months. The pain was constant, dull aching in nature without any radiation and aggravating and relieving factor. There was no history of fever, night sweats, chest pain, hemoptysis, back pain, abdominal swelling, decreased appetite, weight loss, melena, and alteration of bowel habit. He was not suffering from any kind of morbidity. There was no history of similar problem in the past. His family history was not significant. General survey and systemic examinations were unremarkable. Digital rectal examination was also within the normal limit.

**Figure 1:**
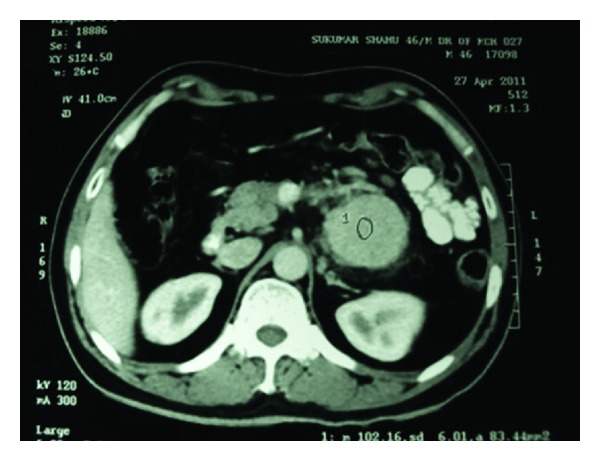
CECT showing homogenous mass in relation to the tail of the pancreas.

Ultrasonography (USG) of whole abdomen showed hypoechoic lesion adjacent to the tail of the pancreas on its posterior aspect on left of abdominal aorta and medial to spleen and kidney which was suggestive of neoplastic lesion in relation to the tail of the pancreas or a retroperitoneal tumor. Contrast enhanced computerized tomography (CECT) of the whole abdomen revealed a homogenous enhanced mass (measuring 4.6 cm × 4.4 cm × 4 cm) behind the tail of pancreas without obliteration of surrounding plane (Figure 1). The margins of the lesion were well circumscribed. CECT did not show any lymphadenopathy, calcification, or ascites and findings of other organs were within normal limit. Colonoscopic examination, chest X-ray, complete hemogram, and blood biochemistry did not show any abnormality. Serological tests for active HIV and Hepatitis B and C and polymerase chain reaction test for herpes simplex virus 8 were negative. The serum IL-6 level was within the normal limit. Tumor markers (CEA and CA 19-9) were not raised. The provisional diagnosis of pancreatic neoplasm was made.

**Figure 2:**
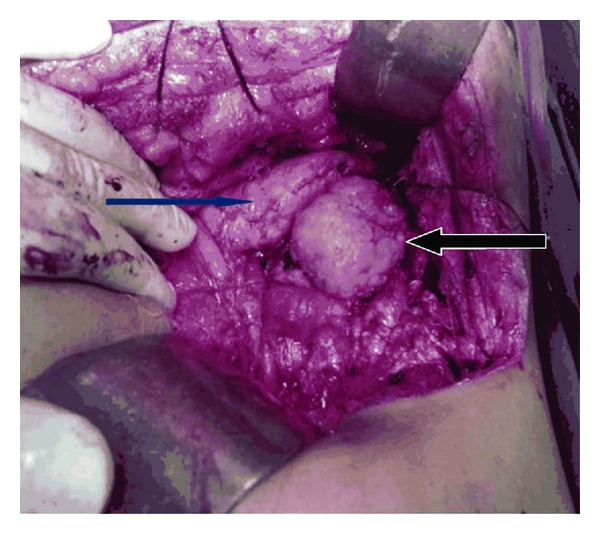
The blue arrow shows pancreas and the black arrow shows Castleman's disease.

The patient underwent exploratory laparotomy through chevron incision. On entering the lesser sac, a 5 cm × 4.5 cm × 4.2 cm firm mass with fibroelastic consistency was noted behind the tail of the pancreas without invasion to the surrounding organs (Figure 2). The pancreas, liver, and other organs appeared to be normal. The mass was enucleated with preservation of other surrounding organs. No enlarged lymph nodes, ascites, or peritoneal/omental metastasis was detected. Postoperative period was uneventful and he was discharged on 10th postoperative day. Histopathological examination of the retroperitoneum mass revealed Castleman's disease of hyaline vascular (angiofollicular) type presenting with characteristic features of onion peel cellular deposition with negative resection margins. On followup for the next one year, the patient was healthy without any feature of recurrence. 

## 3. Discussion 

Castleman's disease (CD) was first reported by Symmers in 1921 [4]. It is more common in the younger age group, although age varies from 8 to 66 years, equally affects males and females [3] Overproduction of interleukin 6 and infection with human herpes virus type 8, HIV, HBV/HCV, and Epstein-Barr and other several mechanisms have been proposed, but etiology is still unclear [5].

CD is classified depending on its distribution in two subtypes—a localized or unicentric subtype with good prognosis and a multicentric subtype with worse prognosis. Histologically it is also classified into three subtypes: hyaline-vascular subtype, plasma cell subtype, and mixed form (hyaline-vascular-plasma cell) subtype [3, 6]. The hyaline-vascular variety is more common (90%) and is usually asymptomatic, whereas the plasma cell subtype is less common, and about 50% of these patients present with anemia, fever, fatigue, hyperglobulinemia, and hypoalbuminemia [7].

CD may occur anywhere along the lymphoid chain [3]. Of the 400 reported cases [8], the majority of the cases involved thorax and neck and few cases were found in abdomen and axilla (12% and 4%, resp.). Among the intra-abdominal lesions, most are located in the pelvic, mesenteric, and perinephric regions [9] but may be found throughout abdomen. Among 195 cases of localized abdominal and retroperitoneal CD, 122 (63%) were in the retroperitoneum and 73 (37%) in the abdominal cavity [10]. Of these 122 lesions localized in the retroperitoneum, 5 (0.25%) cases were found in the peripancreatic region, as in our case report. Unicentric CD in the peripancreatic region is a rare clinical entity. It always possesses a diagnostic challenge to differentiate from pancreatic neoplasm. 

The localized form of CD generally affects younger and healthy patients. It remains usually asymptomatic [11, 12]. Abdominal and retroperitoneal locations may present with symptoms of mass effect on adjacent organs, for example, anorexia, weight loss, vomiting, urinary retention, and abdominal pain. In our case, the patient presented to us with left upper abdominal pain. Systemic or multifocal CD presents with systemic disturbances such as anemia, increased ESR, polyclonal hypergammaglobulinemia, hypoalbuminemia, and thrombocytopenia [11–13]. The symptoms include asthenia, fever, weight loss, splenomegaly, hepatomegaly, generalized lymphadenopathy, peripheral edema, pleural effusion, impaired renal function, and sometimes polyneuropathy. It is also associated with an acquired immunodeficiency syndrome, Kaposi's sarcoma, POEMS syndrome (polyneuropathy, organomegaly, endocrinopathy, M protein, and skin change), and amyloidosis [11–13]. Sometimes CD is detected incidentally as a slow-growing mass. The unicentric hyaline-vascular type of CD rarely shows systemic symptoms (<10%) [3, 14]. The most commonly described (77–91%) symptoms in the literature is a localized [3, 14] and an asymptomatic mass [3, 15]. A recently published series of eight cases of peripancreatic unicentric CD showed that most of the patients were asymptomatic and few cases complained of vague abdominal pain. In that series, the most common histological variety was the hyaline vascular form [16].

Ultrasound and CT or MRI imaging modalities cannot diagnose CD because of the lack of tumor-specific signs but give information about the exact tumor location [7]. Other nonspecific diagnostic abnormalities include anemia, hypoalbuminemia, polyclonal gammopathy, elevated erythrocyte sedimentation rate or C-reactive protein concentration, and proteinuria [17], which is not found in the localized form. Gallium scintigraphy is considered sensitive for diagnosis and detection of the hyaline-vascular type of CD, but in the plasma cell variant, it is debated [18]. Only surgical resection and conventional histological evaluation can give an accurate characterization of this tumor.

Treatment of the unicentric CD (UCD) is the resection, which gives excellent long-term results [6]. Radiotherapy is also considered a treatment option for patients with poor surgical approach or had surgery with incomplete resection [7]. In patients with the multicentric CD, surgical treatment is not beneficial and steroid treatment is the mainstay, with or without chemotherapy [5, 6]. Due to poor prognosis and low rate of survival, the treatment modality of choice for the multicentric disease is yet to established [6]. Our case of UCD got only surgical excision with negative margin.

In conclusion, Castleman's disease is a rare retroperitoneal lump which generally remains asymptomatic. Peripancreatic location of unicentric Castleman's disease is uncommon. As it is difficult to make preoperative diagnosis, CD should be included in the differential diagnosis of pancreatic neoplasm. Enucleation with negative margin claims an excellent five-year survival.
